# Dietary and Physical Activity Behaviors in 2021 and Changes from 2019 to 2021 Among High School Students — Youth Risk Behavior Survey, United States, 2021

**DOI:** 10.15585/mmwr.su7201a9

**Published:** 2023-04-28

**Authors:** Shannon L. Michael, Sherry Everett Jones, Caitlin L. Merlo, Sarah A. Sliwa, Sarah M. Lee, Kelly Cornett, Nancy D. Brener, Tiffany J. Chen, Carmen L. Ashley, Sohyun Park

**Affiliations:** ^1^Division of Population Health, National Center for Chronic Disease Prevention and Health Promotion, CDC; ^2^Division of Adolescent and School Health, National Center for HIV, Viral Hepatitis, STD, and TB Prevention, CDC; ^3^Division of Nutrition, Physical Activity, and Obesity, National Center for Chronic Disease Prevention and Health Promotion, CDC; ^4^McKing Consulting Corporation, Atlanta, Georgia

## Abstract

The fall of 2021 was the first school semester to begin with widespread in-person learning since the COVID-19 pandemic began. Understanding dietary and physical activity behaviors of adolescents during this time can provide insight into potential health equity gaps and programmatic needs in schools and communities. This report uses data from the 2021 national Youth Risk Behavior Survey conducted among a nationally representative sample of U.S. public and private school students in grades 9–12 to update estimates of dietary and physical activity behaviors among U.S. high school students overall and by sex and race and ethnicity. In addition, 2-year comparisons (2019 versus 2021) of these behaviors were examined. In 2021, daily consumption of fruits, vegetables, and breakfast during the past 7 days remained low and decreased overall with specific disparities by sex and race and ethnicity from 2019 to 2021. The overall prevalence of students attending physical education classes daily, exercising to strengthen muscles on ≥3 days/week (i.e., met the guideline for muscle-strengthening activity), and playing on at least one sports team decreased from 2019 to 2021; whereas being physically active for ≥60 minutes/day on all 7 days (i.e., met the guideline for aerobic activity) and meeting both aerobic and muscle-strengthening guidelines remained low but did not change. These findings underscore the need for strategies to increase healthy dietary and physical activity behaviors both in the recovery phase of COVID-19 and longer term.

## Introduction

Healthy dietary and physical activity behaviors provide adolescents with various benefits and are important public health strategies for chronic disease prevention (*1**,**2*). These benefits include supporting healthy growth and development, maintaining a healthy body weight, reducing anxiety, and reducing the risk for developing health conditions (e.g., heart disease or type 2 diabetes) (*1**,**2*). Not having consistent opportunities to practice these health behaviors could negatively affect students’ physical and mental health, which have long-term health implications (*1**–**3*).

From the start of the COVID-19 pandemic in 2020, school and community practices changed to comply with COVID-19 guidance. Such changes included modified meal services and sport schedules, which likely affected opportunities for students to consistently engage in healthy dietary and physical activity behaviors (*4*,*5*). For example, recent studies illustrated that breakfast regularity declined during the pandemic, whereas afternoon and evening snack consumption increased among adolescents (*6*), and homes had more high-calorie snack foods, unhealthy foods (e.g., desserts or sweets), and nonperishable processed foods during the pandemic (*7*). Likewise, adolescent physical activity levels decreased at the beginning of the pandemic because of changes in school- and sports-based programs (*4*,*8*). These pandemic-related effects are concerning because students might not meet key recommendations in the Dietary Guidelines for Americans 2020–2025 for following a healthy eating pattern (*1*) and not reach the duration and frequency of physical activity recommended by the Physical Activity Guidelines for Americans, second edition (*2*).

Despite studies examining dietary and physical activity behaviors of students between March 2020 and July 2021 (*4*–*8*), little is known about these behaviors among U.S. high school students during the fall of 2021 when most of them returned to school in person. This report provides 2021 national estimates of dietary and physical activity behaviors among U.S. high school students overall and by sex and race and ethnicity. This report also compares 2019 with 2021 data overall and by sex and race and ethnicity to identify health disparities magnified during the pandemic. Health professionals, state and local health officials, policymakers, and school leaders can use the findings in this report to highlight the need for school health policies, practices, and programs that promote students’ healthy dietary and physical activity behaviors and their overall physical and mental health during immediate and longer-term pandemic recovery efforts.

## Methods

### Data Source

This report includes data from the 2019 (N = 13,677) and 2021 (N = 17,232) YRBS, a cross-sectional, school-based survey conducted biennially since 1991. Each survey year, CDC collects data from a nationally representative sample of public and private school students in grades 9–12 in the 50 U.S. states and the District of Columbia. Additional information about YRBS sampling, data collection, response rates, and processing is available in the overview report of this supplement (*9*). The prevalence estimates for dietary and physical activity behaviors for the overall study population and by sex, race and ethnicity, grade, and sexual identity are available at https://nccd.cdc.gov/youthonline/App/Default.aspx. The full YRBS questionnaire, data sets, and documentation are available at https://www.cdc.gov/healthyyouth/data/yrbs/index.htm. This activity was reviewed by CDC and was conducted consistent with applicable federal law and CDC policy.*

### Measures

Six dietary variables and five physical activity variables were examined for this report (Table 1). The dietary variables included the following: during the 7 days before the survey, had eaten fruit or drunk 100% fruit juices <1 time/day, had eaten vegetables <1 time/day, had not eaten breakfast on all 7 days (i.e., did not eat breakfast daily), had drunk soda or pop ≥1 time/day (not counting diet soda or diet pop), had drunk a sports drink ≥1 time/day, and had drunk <3 glasses/day of plain water. The physical activity variables included the following: during the 7 days before the survey, had been physically active for a total of ≥60 minutes/day on all 7 days (i.e., met the federal guideline for aerobic activity) (*2*), had exercised to strengthen or tone muscles on ≥3 days (i.e., met the federal guideline for muscle-strengthening activity), had met both aerobic and muscle-strengthening guidelines, had attended physical education classes on all 5 days during an average school week, and had played on ≥1 sports team during the 12 months before the survey.

**TABLE 1:** Question wording and analytic coding for included dietary and physical activity behavior variables — Youth Risk Behavior Survey, United States, 2021

Variable	Question	Response options	Analytic coding
**Poor dietary behaviors**			
Ate fruit or drank 100% fruit juices <1 time/day*	During the past 7 days, how many times did you... • drink 100% fruit juices such as orange juice, apple juice, or grape juice? (Do not count punch, Kool-Aid, sports drinks, or other fruit-flavored drinks.) • eat fruit? (Do not count fruit juice.)	I did not [drink 100% fruit juice]/[eat fruit] during the past 7 days, 1–3 times during the past 7 days, 4–6 times during the past 7 days, 1 time/day, 2 times/day, 3 times/day, or ≥4 times/day	<1 time/day versus ≥1 time/day
Ate vegetables <1 time/day*	During the past 7 days, how many times did you eat... • green salad? • potatoes? (Do not count French fries, fried potatoes, or potato chips.) • carrots? • other vegetables? (Do not count green salad, potatoes, or carrots.)	I did not eat [green salad]/[potatoes]/[carrots]/[other vegetables] during the past 7 days, 1–3 times during the past 7 days, 4–6 times during the past 7 days, 1 time/day, 2 times/day, 3 times/day, or ≥4 times/day	<1 time/day versus ≥1 time/day
Did not eat breakfast daily	During the past 7 days, on how many days did you eat breakfast?	0 days, 1 day, 2 days, 3 days, 4 days, 5 days, 6 days, or 7 days	≤6 days versus 7 days
Drank soda or pop ≥1 time/day	During the past 7 days, how many times did you drink a can, bottle, or glass of soda or pop, such as Coke, Pepsi, or Sprite? (Do not count diet soda or diet pop.)	I did not drink soda or pop during the past 7 days, 1–3 times during the past 7 days, 4–6 times during the past 7 days, 1 time/day, 2 times/day, 3 times/day, or ≥4 times/day	≥1 time/day versus <1 time/day
Drank a sports drink ≥1 time/day	During the past 7 days, how many times did you drink a can, bottle, or glass of a sports drink, such as Gatorade or Powerade? (Do not count low-calorie sports drinks such as Propel or G2.)	I did not drink sports drinks during the past 7 days, 1–3 times during the past 7 days, 4–6 times during the past 7 days, 1 time/day, 2 times/day, 3 times/day, or ≥4 times/day	≥1 time/day versus <1 time/day
Drank <3 glasses/day of plain water	During the past 7 days, how many times did you drink a bottle or glass of plain water? (Count tap, bottled, and unflavored sparkling water.)	I did not drink water during the past 7 days, 1–3 times during the past 7 days, 4–6 times during the past 7 days, 1 time/day, 2 times/day, 3 times/day, or ≥4 times/day	<3 times/day versus ≥3 times/day
**Physical activity behaviors**			
Were physically active for a total of ≥60 minutes/day on all 7 days (i.e., met the guideline for aerobic activity)	During the past 7 days, on how many days were you physically active for at least 60 minutes per day? (Add up all the time you spent in any kind of physical activity that increased your heart rate and made you breathe hard some of the time.)	0 days, 1 day, 2 days, 3 days, 4 days, 5 days, 6 days, or 7 days	7 days versus <7 days
Did exercises to strengthen or tone muscles on ≥3 days (i.e., met the guideline for muscle-strengthening activity)	During the past 7 days, on how many days did you do exercises to strengthen or tone your muscles (e.g., push-ups, sit-ups, or weightlifting)?	0 days, 1 day, 2 days, 3 days, 4 days, 5 days, 6 days, or 7 days	≥3 days versus <3 days
Met both aerobic and muscle-strengthening guidelines	[See “were physically active for a total of ≥60 minutes/day on all 7 days” and “did exercises to strengthen or tone muscles on ≥3 days.”]	NA	Physically active for ≥60 minutes/day on all 7 days and did exercises to strengthen or tone muscles on ≥3 days versus physically active for <60 minutes/day on all 7 days or did exercises to strengthen or tone muscles on <3 days
Attended physical education classes on all 5 days	In an average week when you are in school, on how many days do you go to physical education classes?	0 days, 1 day, 2 days, 3 days, 4 days, or 5 days	5 days versus <5 days
Played on ≥1 sports team	During the past 12 months, on how many sports teams did you play? (Count any teams run by your school or community groups.)	0 teams, 1 team, 2 teams, or ≥3 teams	≥1 team versus <1 team

**Abbreviation:** NA = not applicable.

* This variable comprised more than one question. The responses of the questions were summed and then dichotomized to reflect <1 time/day versus ≥1 time/day.

Student demographic characteristics examined included sex (female or male) and race and ethnicity. Students were classified into seven racial and ethnic categories including American Indian or Alaska Native (AI/AN), Asian, Black or African American (Black), Hispanic or Latino (Hispanic), Native Hawaiian or other Pacific Islander (NH/OPI), White, and students who were two or more races (multiracial). (Persons of Hispanic origin might be of any race but are categorized as Hispanic; all racial groups are non-Hispanic.) Grade was also included in the regression model.

### Analysis

For each behavior, the 2021 prevalence and 95% CIs were calculated overall and for each sex and race and ethnicity group. Statistically significant pairwise differences by sex and race and ethnicity were determined by *t*-tests with Taylor series linearization, as were comparisons between 2019 and 2021. Differences between prevalence estimates were considered statistically significant if the *t*-test p value was <0.05. Only statistically significant findings are described.

Using national YRBS data from 2021, four logistic regression models examined the association between: 1) having played on ≥1 sports team and being physically active for ≥60 minutes/day on all 7 days, 2) having played on ≥1 sports team and having met both aerobic and muscle-strengthening guidelines, 3) having attended physical education classes on all 5 days and being physically active for ≥60 minutes/day on all 7 days, and 4) having attended physical education classes on all 5 days and having met both aerobic and muscle-strengthening guidelines. These models controlled for sex, race and ethnicity, and grade. Results from the analyses are reported as adjusted prevalence ratios (APRs) with 95% CIs. APRs were considered statistically significant if the 95% CI did not include 1.0. Prevalence estimates with a denominator <30 were considered statistically unreliable and therefore were suppressed (*9*).

## Results

### Dietary Behaviors

In 2021, 47.1% of students had eaten fruit or drunk 100% fruit juices <1 time/day, 45.3% had eaten vegetables <1 time/day, 75.0% had not eaten breakfast daily, 14.7% had drunk sugar-sweetened soda or pop ≥1 time/day, 11.2% had drunk a sports drink ≥1 time/day, and 44.2% had drunk <3 glasses/day of plain water (Table 2).

**TABLE 2:** Percentage of high school students* with poor dietary behaviors,^†^ by sex and race and ethnicity — Youth Risk Behavior Survey, United States, 2021

Characteristic	Ate fruit or drank 100% fruit juices <1 time/day^§^ % (95% CI)	Ate vegetables <1 time/day^¶^ % (95% CI)	Did not eat breakfast daily** % (95% CI)	Drank sugar-sweetened soda or pop ≥1 time/day^††^ % (95% CI)	Drank a sports drink ≥1 time/day^§§^ % (95% CI)	Drank <3 glasses/day of plain water^¶¶^ % (95% CI)
**Overall**	**47.1 (45.6–48.5)**	**45.3 (42.7–47.9)**	**75.0 (73.1–76.7)**	**14.7 (13.4–16.2)**	**11.2 (9.6–12.9)**	**44.2 (41.9–46.6)**
**Sex**						
Female	50.5 (48.3–52.8)	45.0 (42.8–47.3)	80.1 (77.8–82.2)	12.7 (10.7–14.9)	8.4 (6.6–10.6)	46.1 (43.3–49.0)
Male	43.6 (41.9–45.4)	45.2 (41.9–48.6)	69.9 (67.9–71.9)	16.5 (15.3–17.7)	13.6 (11.8–15.6)	42.2 (39.8–44.7)
Race and ethnicity***						
American Indian or Alaska Native	49.1 (36.2–62.2)	37.4 (23.6–53.6)	77.9 (68.7–85.0)	22.8 (12.7–37.5)	21.0 (13.5–31.4)	37.6 (24.7–52.6)
Asian	40.6 (31.0–51.0)	30.4 (21.4–41.2)	61.9 (55.9–67.5)	5.4 (4.1–6.9)	3.9 (2.3–6.5)	28.1 (21.9–35.3)
Black or African American	47.0 (44.1–50.0)	55.7 (52.5–59.0)	83.8 (81.0–86.2)	15.1 (12.5–18.2)	18.7 (14.9–23.2)	48.1 (44.6–51.7)
Native Hawaiian or other Pacific Islander	48.8 (35.8–62.0)	59.0 (40.6–75.1)	80.0 (66.6–88.9)	18.2 (8.3–35.4)	—^†††^	41.3 (28.6–55.4)
White	46.9 (44.3–49.6)	41.7 (38.8–44.7)	72.4 (69.7–75.0)	15.8 (13.8–18.2)	10.2 (8.6–12.1)	46.0 (43.5–48.6)
Hispanic or Latino	48.2 (46.5–49.9)	50.7 (47.8–53.5)	77.7 (75.1–80.2)	14.0 (12.4–15.9)	11.5 (10.5–12.5)	43.1 (38.8–47.4)
Multiracial	48.0 (43.7–52.3)	41.1 (36.4–46.1)	78.1 (70.8–83.9)	12.5 (9.8–15.9)	9.6 (7.6–12.1)	40.8 (35.9–45.9)

* N = 17,232 respondents. Because the state and local questionnaires differ by jurisdiction, students in these schools were not asked all national YRBS questions. Therefore, the total number (N) of students answering each question varied. Percentages in each category are calculated on the known data.

^†^ Refer to Table 1 for variable definitions.

^§^ On the basis of *t*-test analysis with Taylor series linearization (p<0.05). Female students significantly different from male students. No significant differences by race and ethnicity.

^¶^ On the basis of *t*-test analysis with Taylor series linearization (p<0.05). No significant differences by sex. American Indian or Alaska Native (AI/AN) students significantly different from Black or African American (Black) students; Asian students significantly different from Black, Hispanic or Latino (Hispanic), Native Hawaiian and other Pacific Islander (NH/OPI), White, and multiracial students; Black students significantly different from Hispanic, White, and multiracial students; Hispanic students significantly different from White and multiracial students; and NH/OPI significantly different from White students.

** On the basis of *t*-test analysis with Taylor series linearization (p<0.05). Female students significantly different from male students. Asian students significantly different from AI/AN, Black, Hispanic, NH/OPI, White, and multiracial students; Black students significantly different from Hispanic, White, and multiracial students; and Hispanic students significantly different from White students.

^††^ On the basis of *t*-test analysis with Taylor series linearization (p<0.05). Female students significantly different from male students. AI/AN significantly different from Asian students; Asian students significantly different from Black, Hispanic, White, and multiracial students; and White students significantly different from multiracial students.

^§§^ On the basis of *t*-test analysis with Taylor series linearization (p<0.05). Female students significantly different from male students. AI/AN students significantly different from Asian, Hispanic, White, and multiracial students; Asian students significantly different from Black, Hispanic, White, and multiracial students; and Black students significantly different from Hispanic, White, and multiracial students.

^¶¶^ On the basis of *t*-test analysis with Taylor series linearization (p<0.05). Female students significantly different from male students. Asian students significantly different from Black, Hispanic, White, and multiracial students; and Black students significantly different from Hispanic and multiracial students.

*** Persons of Hispanic origin might be of any race but are categorized as Hispanic; all racial groups are non-Hispanic.

^†††^ Prevalence estimates with a denominator <30 were considered statistically unreliable and therefore were suppressed.

Dietary behaviors varied by demographic characteristics. A higher percentage of female than male students had eaten fruit or drunk 100% fruit juices <1 time/day (50.5% versus 43.6%), had not eaten breakfast daily (80.1% versus 69.9%), and had drunk <3 glasses/day of plain water (46.1% versus 42.2%). In contrast, a higher percentage of male than female students had drunk sugar-sweetened soda or pop ≥1 time/day (16.5% versus 12.7%) and had drunk a sports drink ≥1 time/day (13.6% versus 8.4%). Although certain exceptions were observed, the prevalence of these poor dietary behaviors was lower among Asian students, but higher among Black students, than among students from other racial and ethnic groups.

During 2019–2021, increases occurred for three of the poor dietary behaviors examined (Table 3). The percentage of students who had eaten fruit or drunk 100% fruit juices <1 time/day increased overall and among female, male, Hispanic, and White students. The percentage of students who had eaten vegetables <1 time/day also increased overall and among female and White students. In addition, the percentage of students who had not eaten breakfast daily increased overall and among female, male, Black, Hispanic, and White students.

**TABLE 3:** Percentage of high school students* with poor dietary behaviors,^†^ by survey year, sex, and race and ethnicity — Youth Risk Behavior Survey, United States, 2019 and 2021

Behavior	2019	2021	Change from 2019 to 2021^§^
**Ate fruit or drank 100% fruit juices <1 time/day**			
**Overall**	**41.8**	**47.1**	**Increased**
**Sex**			
Female	43.0	50.5	Increased
Male	40.6	43.6	Increased
**Race and ethnicity^¶^**			
American Indian or Alaska Native	44.0	49.1	No change
Asian	33.5	40.6	No change
Black or African American	47.8	47.0	No change
Native Hawaiian or other Pacific Islander	51.8	48.8	No change
White	42.1	46.9	Increased
Hispanic or Latino	39.5	48.2	Increased
Multiracial	41.7	48.0	No change
**Ate vegetables <1 time/day**			
**Overall**	**40.7**	**45.3**	**Increased**
**Sex**			
Female	40.4	45.0	Increased
Male	41.1	45.2	No change
**Race and ethnicity^¶^**			
American Indian or Alaska Native	38.5	37.4	No change
Asian	22.3	30.4	No change
Black or African American	54.8	55.7	No change
Native Hawaiian or other Pacific Islander	40.9	59.0	No change
White	35.5	41.7	Increased
Hispanic or Latino	46.8	50.7	No change
Multiracial	41.4	41.1	No change
**Did not eat breakfast daily**			
**Overall**	**66.9**	**75.0**	**Increased**
**Sex**			
Female	71.5	80.1	Increased
Male	62.4	69.9	Increased
**Race and ethnicity^¶^**			
American Indian or Alaska Native	82.3	77.9	No change
Asian	52.5	61.9	No change
Black or African American	72.0	83.8	Increased
Native Hawaiian or other Pacific Islander	66.3	80.0	No change
White	65.5	72.4	Increased
Hispanic or Latino	67.3	77.7	Increased
Multiracial	77.4	78.1	No change
**Drank sugar-sweetened soda or pop ≥1 time/day**			
**Overall**	**15.1**	**14.7**	**No change**
**Sex**			
Female	11.7	12.7	No change
Male	18.2	16.5	No change
**Race and ethnicity^¶^**			
American Indian or Alaska Native	25.0	22.8	No change
Asian	4.6	5.4	No change
Black or African American	16.9	15.1	No change
Native Hawaiian or other Pacific Islander	10.3	18.2	No change
White	15.2	15.8	No change
Hispanic or Latino	16.1	14.0	No change
Multiracial	13.4	12.5	No change
**Drank a sports drink ≥1 time/day**			
**Overall**	**10.6**	**11.2**	**No change**
**Sex**			
Female	7.1	8.4	No change
Male	14.0	13.6	No change
**Race and ethnicity^¶^**			
American Indian or Alaska Native	27.2	21.0	No change
Asian	2.1	3.9	No change
Black or African American	15.6	18.7	No change
Native Hawaiian or other Pacific Islander	9.4	—**	—
White	9.3	10.2	No change
Hispanic or Latino	11.9	11.5	No change
Multiracial	13.5	9.6	No change
**Drank <3 glasses/day of plain water**			
**Overall**	**44.6**	**44.2**	**No change**
**Sex**			
Female	44.1	46.1	No change
Male	45.0	42.2	No change
**Race and ethnicity^¶^**			
American Indian or Alaska Native	49.4	37.6	No change
Asian	34.4	28.1	No change
Black or African American	54.8	48.1	No change
Native Hawaiian or other Pacific Islander	38.5	41.3	No change
White	44.2	46.0	No change
Hispanic or Latino	44.2	43.1	No change
Multiracial	38.6	40.8	No change

* 2019: N = 13,677 respondents; 2021: N = 17,232 respondents. Because the state and local questionnaires differ by jurisdiction, students in these schools were not asked all national YRBS questions. Therefore, the total number (N) of students answering each question varied. Percentages in each category are calculated on the known data.

^†^ Refer to Table 1 for variable definitions.

^§^ On the basis of *t*-test analysis with Taylor series linearization (p<0.05). An increase indicates a worsening of dietary behavior.

^¶^ Persons of Hispanic or Latino (Hispanic) origin might be of any race but are categorized as Hispanic; all racial groups are non-Hispanic.

** Prevalence estimates with a denominator <30 were considered statistically unreliable and therefore were suppressed.

### Physical Activity Behaviors

In 2021, 23.9% of students had been physically active for ≥60 minutes/day on all 7 days, 44.9% had exercised to strengthen or tone their muscles ≥3 days/week, 16.0% had met both aerobic and muscle-strengthening guidelines, 19.0% had attended physical education classes on all 5 days, and 49.1% had played on ≥1 sports team (Table 4).

**TABLE 4:** Percentage of high school students* with physical activity behaviors,^†^ by sex and race and ethnicity — Youth Risk Behavior Survey, United States, 2021

Characteristic	Were physically active for a total of ≥60 minutes/day on all 7 days^§^ % (95% CI)	Did exercises to strengthen or tone muscles on ≥3 days^¶^ % (95% CI)	Met both aerobic and muscle-strengthening guidelines** % (95% CI)	Went to physical education classes on all 5 days^††^ % (95% CI)	Played on ≥1 sports team^§§^ % (95% CI)
**Overall**	**23.9 (22.8–25.0)**	**44.9 (42.5–47.2)**	**16.0 (14.2–17.9)**	**19.0 (15.7–22.7)**	**49.1 (46.3–51.8)**
**Sex**					
Female	15.7 (14.1–17.4)	32.3 (29.7–35.1)	8.8 (7.3–10.6)	16.7 (13.4–20.6)	46.4 (43.4–49.4)
Male	31.7 (30.2–33.2)	56.6 (54.4–58.8)	22.9 (20.5–25.4)	21.1 (17.2–25.6)	52.0 (49.1–55.0)
**Race and ethnicity^¶¶^**					
American Indian or Alaska Native	40.0 (22.5–60.3)	54.8 (39.2–69.5)	29.9 (15.1–50.5)	23.0 (14.7–34.2)	52.8 (41.8–63.6)
Asian	19.4 (14.6–25.3)	41.7 (35.2–48.5)	13.5 (7.9–22.1)	9.6 (5.7–15.6)	45.0 (33.7–56.8)
Black or African American	19.7 (17.5–22.0)	40.7 (36.1–45.4)	10.8 (8.4–13.7)	19.6 (13.9–27.0)	47.2 (43.1–51.3)
Native Hawaiian or other Pacific Islander	23.2 (16.1–32.2)	43.2 (31.6–55.6)	15.0 (10.9–20.4)	15.9 (8.4–28.2)	50.6 (32.6–68.4)
White	27.7 (25.1–30.4)	47.0 (43.3–50.6)	18.6 (15.8–21.8)	19.0 (15.0–23.6)	55.3 (51.4–59.2)
Hispanic or Latino	18.9 (17.3–20.5)	44.2 (41.9–46.5)	13.5 (11.8–15.4)	21.0 (17.4–25.2)	39.4 (36.7–42.1)
Multiracial	21.3 (17.8–25.2)	39.4 (35.1–43.7)	13.5 (10.5–7.1)	16.5 (10.5–24.9)	48.8 (43.8–53.8)

* N = 17,232 respondents. Because the state and local questionnaires differ by jurisdiction, students in these schools were not asked all national YRBS questions. Therefore, the total number (N) of students answering each question varied. Percentages in each category are calculated on the known data.

^†^ Refer to Table 1 for variable definitions.

^§^ On the basis of *t*-test analysis with Taylor series linearization (p<0.05). Female students significantly different from male students. American Indian or Alaska Native (AI/AN) students significantly different from Asian, Black or African American (Black), and Hispanic or Latino (Hispanic) students; and Asian, Black, Hispanic, and multiracial students significantly different from White students.

^¶^ On the basis of *t*-test analysis with Taylor series linearization (p<0.05). Female students significantly different from male students. White students significantly different from multiracial students.

** On the basis of *t*-test analysis with Taylor series linearization (p<0.05). Female students significantly different from male students. AI/AN students significantly different from Asian and Black students; Black students significantly different from Hispanic and White students; and Hispanic and multiracial students significantly different from White students.

^††^ On the basis of *t*-test analysis with Taylor series linearization (p<0.05). Female students significantly different from male students. Asian students significantly different from AI/AN, Black, Hispanic, White, and multiracial students.

^§§^ On the basis of *t*-test analysis with Taylor series linearization (p<0.05). Female students significantly different from male students. AI/AN students significantly different from Hispanic students; Black students significantly different from Hispanic and White students; Hispanic students significantly different from White and multiracial students; and White students significantly different from multiracial students.

^¶¶^ Persons of Hispanic origin might be of any race but are categorized as Hispanic; all racial groups are non-Hispanic.

Physical activity behaviors varied by demographic characteristics. A higher percentage of male than female students had been physically active for ≥60 minutes/day on all 7 days (31.7% versus 15.7%), had exercised to strengthen or tone their muscles on ≥3 days/week (56.6% versus 32.3%), had met both aerobic and muscle-strengthening guidelines (22.9% versus 8.8%), had attended physical education classes on all 5 days (21.1% versus 16.7%), and had played on ≥1 sports team (52.0% versus 46.4%).

Differences by race and ethnicity illustrated no clear pattern across all the physical activity behaviors. For example, the prevalence of being physically active for a total of ≥60 minutes/day on all 7 days was higher among AI/AN students than among Asian, Black, and Hispanic students. Whereas, the prevalence of meeting both aerobic and muscle-strengthening guidelines, and playing on ≥1 sports team was higher among White students than among Black, Hispanic, and multiracial students.

During 2019–2021, decreases occurred overall for three of the five physical activity behaviors examined (Table 5). The percentage of students who had exercised to strengthen or tone their muscles on ≥3 days/week decreased overall and among female, Black, NH/OPI, and multiracial students. The percentage of students who had attended physical education classes on all 5 days decreased overall and among male, Asian, Hispanic, and NH/OPI students. The percentage of students who had played on ≥1 sports team decreased overall and among female, male, Black, Hispanic, White, and multiracial students. In addition, although the percentage of students who had met the aerobic and muscle-strengthening guidelines did not change overall, the percentage decreased among NH/OPI students.

**TABLE 5:** Percentage of high school students* with physical activity behaviors,^†^ by survey year, sex, and race and ethnicity — Youth Risk Behavior Survey, United States, 2019 and 2021

Behavior	2019	2021	Change from 2019 to 2021^§^
**Were physically active for a total of ≥60 minutes/day on all 7 days**			
**Overall**	**23.2**	**23.9**	**No change**
**Sex**			
Female	15.4	15.7	No change
Male	30.9	31.7	No change
**Race and ethnicity^¶^**			
American Indian or Alaska Native	26.5	40.0	No change
Asian	15.3	19.4	No change
Black or African American	21.1	19.7	No change
Native Hawaiian or other Pacific Islander	37.0	23.2	No change
White	25.6	27.7	No change
Hispanic or Latino	20.9	18.9	No change
Multiracial	21.5	21.3	No change
**Did exercises to strengthen or tone muscles on ≥3 days**			
**Overall**	**49.5**	**44.9**	**Decreased**
**Sex**			
Female	39.7	32.3	Decreased
Male	59.0	56.6	No change
**Race and ethnicity^¶^**			
American Indian or Alaska Native	53.1	54.8	No change
Asian	42.4	41.7	No change
Black or African American	47.0	40.7	Decreased
Native Hawaiian or other Pacific Islander	66.3	43.2	Decreased
White	50.8	47.0	No change
Hispanic or Latino	48.1	44.2	No change
Multiracial	51.5	39.4	Decreased
**Met both aerobic and muscle-strengthening guidelines**			
**Overall**	**16.5**	**16.0**	**No change**
**Sex**			
Female	10.1	8.8	No change
Male	23.1	22.9	No change
**Race and ethnicity^¶^**			
American Indian or Alaska Native	19.1	29.9	No change
Asian	8.5	13.5	No change
Black or African American	13.4	10.8	No change
Native Hawaiian or other Pacific Islander	34.9	15.0	Decreased
White	18.4	18.6	No change
Hispanic or Latino	16.0	13.5	No change
Multiracial	13.6	13.5	No change
**Went to physical education classes on all 5 days**			
**Overall**	**25.9**	**19.0**	**Decreased**
**Sex**			
Female	22.8	16.7	No Change
Male	28.9	21.1	Decreased
**Race and ethnicity^¶^**			
American Indian or Alaska Native	22.6	23.0	No change
Asian	27.3	9.6	Decreased
Black or African American	23.8	19.6	No change
Native Hawaiian or other Pacific Islander	41.6	15.9	Decreased
White	24.3	19.0	No change
Hispanic or Latino	29.9	21.0	Decreased
Multiracial	25.6	16.5	No change
**Played on ≥1 sports team**			
**Overall**	**57.4**	**49.1**	**Decreased**
**Sex**			
Female	54.6	46.4	Decreased
Male	60.2	52.0	Decreased
**Race and ethnicity^¶^**			
American Indian or Alaska Native	48.6	52.8	No change
Asian	46.5	45.0	No change
Black or African American	56.1	47.2	Decreased
Native Hawaiian or other Pacific Islander	64.7	50.6	No change
White	62.0	55.3	Decreased
Hispanic or Latino	51.6	39.4	Decreased
Multiracial	57.8	48.8	Decreased

* 2019: N = 13,677 respondents; 2021: N = 17,232 respondents. Because the state and local questionnaires differ by jurisdiction, students in these schools were not asked all national YRBS questions. Therefore, the total number (N) of students answering each question varied. Percentages in each category are calculated on the known data.

^†^ Refer to Table 1 for variable definitions.

^§^ On the basis of *t*-test analysis with Taylor series linearization (p<0.05). A decrease indicates a worsening of the physical activity behavior.

^¶^ Persons of Hispanic or Latino (Hispanic) origin might be of any race but are categorized as Hispanic; all racial groups are non-Hispanic.

### Associations Between Physical Activity Behaviors

The findings in this report illustrated decreases from 2019 to 2021 in both the prevalence of students who had attended physical education classes on all 5 days and students who had played on ≥1 sports team but no changes in the prevalence estimates of having been physically active for ≥60 minutes/day on all 7 days or having met both aerobic and muscle-strengthening guidelines. These observations warranted an examination of the potential effect of physical education and sports participation on being physically active and meeting both guidelines.

In 2021, after adjusting for sex, race and ethnicity, and grade, students who played on ≥1 sports team compared with those who did not were 2.6 times more likely to be physically active for ≥60 minutes/day on all 7 days (APR = 2.6; CI = 2.4–2.8) and 3.6 times more likely to have met both aerobic and muscle-strengthening guidelines (APR = 3.6; CI = 3.3–4.0). Similarly, students who attended physical education classes on all 5 days compared with those who did not were 1.8 times more likely to be physically active for ≥60 minutes/day on all 7 days (APR = 1.8; CI = 1.5–2.0) and 2.1 times more likely to have met both aerobic and muscle-strengthening guidelines (APR = 2.1; CI = 1.7–2.5).

## Discussion

Although healthy dietary and physical activity behaviors are important for adolescents’ overall physical health, this study found that none of the 11 behaviors examined in this report have improved since 2019. Certain dietary and physical activity behaviors have worsened overall and for certain sex and racial and ethnic groups. These findings are particularly concerning because of the association between poor dietary behaviors and insufficient physical activity and numerous chronic health conditions and poor mental health (*1*–*3*). Understanding current dietary and physical activity behaviors among students and comparing them to prepandemic data can identify areas of high need and be used to influence longer-term physical and mental health outcomes through primary chronic disease prevention strategies.

Overall, these findings illustrate that certain students are not engaging in healthy dietary behaviors. Specifically, in 2021, consumption of fruits, vegetables, and daily breakfast remained low with certain disparities by sex and race and ethnicity, and these behaviors worsened overall from 2019 to 2021. Multiple factors could have contributed to these changes. For example, during the pandemic, certain students might have shifted away from healthy foods in favor of unhealthy alternatives to alleviate stress (*10*).

Although no differences were observed from 2019 to 2021 in consuming sports drinks ≥1 time/day, consuming soda ≥1 time/day, or consuming water <3 times/day, consumption of sugary drinks remained elevated in 2021. This poor dietary behavior is concerning because of its association with chronic diseases (*1*).

In 2021, with the exception of low fruit consumption, all poor dietary behaviors were lower among Asian students compared with students from other racial and ethnic groups. A previous study illustrated similar results for Asian students for sugar-sweetened beverage consumption but no significant findings for fruit and vegetable intake; breakfast consumption was not examined in that study (*11*). This study also found that three of the poor dietary behaviors (i.e., ate vegetables <1 time/day, had not eaten breakfast on any of the past 7 days, and drank a sports drink ≥1 time/day) were higher among Black students compared with Hispanic, White, and multiracial students. This observation is consistent with findings from 2019 (*12*).

The prevalence of all five physical activity behaviors was below 50%, and three of these behaviors decreased from 2019 to 2021. This observation was not surprising because of changes in adolescents’ school and extracurricular schedules as a result of the COVID-19 pandemic. Being physically active for ≥60 minutes/day on all 7 days (i.e., meeting guideline for aerobic activity) and meeting both aerobic and muscle-strengthening guidelines did not change from 2019 to 2021, which is inconsistent with findings from the beginning of the pandemic illustrating that physical activity decreased (*4*). However, the prevalence estimates for 2021 are still troubling, with less than one fourth (23.9%) of students getting the recommended ≥60 minutes of physical activity daily and only 16.0% meeting both aerobic and muscle-strengthening guidelines. Not meeting national physical activity guidelines means that students are not receiving the multiple physical and mental health benefits of physical activity (e.g., reducing stress, anxiety, and depression) and preventing various chronic disease risk factors (*2*).

The results in this report indicate decreases from 2019 to 2021 in physical education class attendance and sports team participation overall for certain sex and racial and ethnic groups. Both of these physical activity behaviors were affected by school closures during the COVID-19 pandemic. It is unclear why these two physical activity opportunities declined although meeting guidelines did not; however, the results of the logistic regression in this study illustrated that students who attend physical education classes daily or participate on a sports team are more likely to get ≥60 minutes of daily physical activity and meet the guidelines, indicating that opportunities for physical activity in and out of school are both important for meeting guidelines. Physical education classes and sports opportunities are also critical for developing social and emotional learning competencies (e.g., social interaction skills, communication skills, teamwork, and goal setting) as well as fostering school connectedness (https://www.shapeamerica.org/standards/guidelines/sel-crosswalk.aspx). School and other types of COVID-19 closures also might have maintained or exacerbated inequities related to accessing physical activity because students might have stayed at or close to their home and neighborhood with varying levels of safety and access to physical activity supports (*13*).

Similar to dietary behaviors, differences across racial and ethnic groups were inconsistent for the physical activity behaviors. However, this study illustrates that being physically active for ≥60 minutes/day on all 7 days, meeting both aerobic and muscle-strengthening guidelines, and playing on a sports team were higher among White students compared with Black, Hispanic, and multiracial students. A recent study had similar findings, indicating that White female adolescents had higher physical activity participation compared with Black, Hispanic, and other minority female students (*14*). Other differences across race and ethnicity found in this study warrant further investigation to determine what factors supported higher prevalence of physical activity behaviors among certain groups.

## Limitations

General limitations for the 2021 YRBS are available in the overview report of this supplement (*9*). The findings in this report are subject to at least four additional limitations. First, the national YRBS collects data on frequency of consumption rather than amount; therefore, these data cannot directly determine whether students are meeting specific dietary recommendations. Second, individual measures of socioeconomic status are not accounted for and are known to be associated with dietary consumption and physical activity opportunities (*8*,*15*). Third, this study did not investigate how these behaviors differed by sex within race and ethnicity to further examine health disparities. Finally, specific student experiences during COVID-19 are unknown (e.g., the extent of remote learning, school closure, and community burden of COVID-19). Therefore, quantifying the effect of COVID-19 is limited.

## Future Directions

Schools face multiple priorities, including addressing mental health issues, mitigating learning loss among students, and offering opportunities for students to learn about and practice health behaviors. These priorities do not need to compete. Ensuring regular access to school-based physical activity and school meals that meet U.S. Department of Agriculture nutrition standards support students’ health and readiness to learn (*2*,*16*). For example, schools can address poor dietary behaviors among high school students by encouraging participation in the National School Lunch and School Breakfast Programs and providing multiple opportunities for students to access breakfast, including Grab and Go and Second Chance models (https://frac.org/wp-content/uploads/how_it_works_bic_fact_sheet.pdf; https://fns-prod.azureedge.us/sites/default/files/resource-files/SBPfactsheet.pdf) that do not require students to arrive early to eat in the cafeteria. In addition, the Community Preventive Services Task Force recommends school-based gardening programs combined with nutrition education as a strategy to increase vegetable consumption (https://www.thecommunityguide.org/sites/default/files/assets/Nutrition-Gardening-Fruit-Vegetable-Consumption-Children-508.pdf#:~:text=The Community Preventive Services Task Force recommends school-based,increase children%E2%80%99s vegetable consumption. Rationale Basis of Finding).

Schools are also uniquely suited to provide students with multiple opportunities for physical activity participation. The actions of schools can be supported by other community strategies to increase physical activity promoted by Active People, Healthy Nation, an initiative led by CDC (https://www.cdc.gov/physicalactivity/activepeoplehealthynation/index.html). Further, implementing a Comprehensive School Physical Activity Program (CSPAP) increases opportunities for students to be physically active before, during, and after school, and can be tailored based on available resources, interests, time allotments, and community support (https://www.cdc.gov/healthyschools/physicalactivity/index.htm). A CSPAP approach enables schools to engage community partners, staff members, families, and before- and after-school leaders to increase the total amount of physical activity access for adolescents throughout the day.

## Conclusion

Certain poor dietary behaviors (e.g., skipping breakfast and infrequent consumption of fruits and vegetables) appear to have worsened during the pandemic, and certain students continue to fall short of recommended levels of physical activity. Understanding current dietary and physical activity behaviors among high school students nationwide can support schools, communities, and families to make decisions about strategies needed to improve these behaviors during the pandemic recovery phase and beyond.

## Footnotes

* 45 C.F.R. part 46.102(l)(2), 21 C.F.R. part 56; 42 U.S.C. §241(d); 5 U.S.C. §552a; 44 U.S.C. §3501 et seq.
